# Implementing CYP2C19-guided clopidogrel therapy: a scoping review of pharmacogenomic testing services

**DOI:** 10.1038/s41397-025-00371-4

**Published:** 2025-04-25

**Authors:** Tark J. Patel, Eman Wehbe, Stephen Hughes, Asad E. Patanwala, Leonard Kritharides, Sean Lal, Sanjay Patel, Sophie L. Stocker

**Affiliations:** 1 School of Pharmacy, Faculty of Medicine and Health, The University of Sydney, Sydney, NSW Australia; 2 Department of Pharmacy, Royal Prince Alfred Hospital, Sydney, NSW Australia; 3 ANZAC Research Institute, Concord Repatriation General Hospital, University of Sydney, Sydney, NSW Australia; 4 Cardiology Department, Concord Repatriation General Hospital, University of Sydney, Sydney, NSW Australia; 5 School of Medical Sciences, Faculty of Medicine and Health, The University of Sydney, Sydney, Australia; 6 Department of Cardiology, Royal Prince Alfred Hospital, Sydney, NSW Australia; 7 Centre for Heart Failure and Diseases of the Aorta, The Baird Institute, Sydney, NSW Australia; 8 Heart Research Institute, Sydney, NSW Australia; 9 Department of Clinical Pharmacology and Toxicology, St Vincent’s Hospital, Darlinghurst, NSW Australia; 10 School of Clinical Medicine, St Vincent’s Clinical Campus, The University of New South Wales, Kensington, NSW Australia

**Keywords:** Health services, Public health

## Abstract

Pharmacogenomic testing for CYP2C19 helps personalise clopidogrel therapy and reduces the risk of experiencing a secondary myocardial infarction in individuals with impaired CYP2C19 function. Routine testing, however, is uncommon and it is proposed that the key requirements and processes of testing services are poorly understood. This scoping review aimed to explore the literature for CYP2C19 testing services for clopidogrel and identify their commonalities to inform the design and delivery of future services. In total, 37 eligible studies describing services across hospital and community settings were retrieved. Key elements of delivery included a multi-disciplinary approach involving physicians and pharmacists, provision of pre-implementation training and education, and electronic communication of test results. Result integration into clinical decision support systems improved the practical application of pharmacogenomic testing. The identification of the key requirements and processes may be used by institutions looking to design and deliver CYP2C19 testing services to guide clopidogrel therapy.

## Introduction

Pharmacogenomic (PGx) testing relates to the identification of genetic variations impacting the response to medicines, allowing for personalised therapy within clinical care [1]. A pharmacogenomic guided approach to prescribing helps improve medication efficacy and safety and reduces adverse effect occurrence [2]. Organisations such as the Clinical Pharmacogenetics Implementation Consortium (CPIC) and the Dutch Pharmacogenetics Working Group (DPWG) provide evidence-based guidelines for drugs affected by pharmacogenomic variations. Together, they provide evidence for over 40 unique gene-drug pairs, including CYP2C19 and clopidogrel [3]. Clopidogrel is an antiplatelet most commonly used for the secondary prevention of a myocardial infarction (MI) following catheter revascularisation [4]. As a pro-drug, it relies on CYP2C19 for bioactivation to its active metabolite for its antiplatelet effects [5]. In individuals who are CYP2C19 poor metabolisers, the effectiveness of clopidogrel is significantly reduced due to lower active metabolite production, increasing the risk of experiencing a subsequent MI. In these cases, treatment with ticagrelor or prasugrel is recommended instead [5]. Thus, pre-emptive pharmacogenomic testing plays an important role in mitigating this risk. Whilst other indications for clopidogrel exist (e.g., ischaemic stroke), the role of genotype-guided therapy in these cases remains unclear [6].

Several guidelines, such as those from Australia and Canada, recommend ticagrelor or prasugrel as the first-line agents part of dual antiplatelet therapy post MI [7, 8]. However, in clinical practice, clopidogrel is more commonly used. For example, in Australia, prasugrel has not been available and the use of ticagrelor has been limited by the tight restrictions required for a prescription to receive public subsidy [9, 10]. Hence, in countries where clopidogrel prescribing remains high, an imperative exists to perform CYP2C19 testing to optimise antiplatelet therapy.

There is a growing body of evidence supporting routine CYP2C19 testing. Studies evaluating the long-term outcomes of clopidogrel therapy based on patient genotypes [11] and comparing conventional clopidogrel treatment with genotype-guided treatment [12] have revealed that CYP2C19 testing significantly reduces the incidence of major adverse cardiovascular events (MACE), especially in individuals who are intermediate and poor metabolisers. Additionally, recent statements from the American Heart Association and National Institute for Health and Care Excellence in the UK have further supported the clinical implementation of CYP2C19 genotyping to guide clopidogrel therapy for indications including percutaneous coronary intervention and transient ischaemic attacks [13, 14]. Thus, it is important these recommendations are followed and a move towards routine clinical use of such services is made.

However, a major barrier to achieving this has been a lack of understanding on the process of establishing and delivering such services [15]. Knowledge of the key requirements and processes underpinning CYP2C19 testing services in relation to clopidogrel therapy can help overcome this barrier and may also facilitate implementation of testing services for other gene-drug pairs into practice. Therefore, this scoping review aims to describe various pharmacogenomic testing services and identify their common elements to inform the design and delivery of CYP2C19 testing services to guide clopidogrel therapy.

## Methods

### Protocol

A scoping review was conducted in accordance with the Preferred Reporting Items for Systematic Reviews and Meta-Analyses extension for Scoping Reviews [16].

### Eligibility criteria

Articles describing a model of a pharmacogenomic testing service within any healthcare setting involving CYP2C19 and clopidogrel as either the sole gene-drug pair or part of a broader catalogue were included. Conference articles, abstracts, articles which did not describe a model of testing and those involving CYP2C19 testing for drugs other than clopidogrel were excluded. Articles that did not indicate CYP2C19 and clopidogrel to be a gene-drug pair of focus were also excluded.

### Search strategy

An initial search was carried out via Scopus from database inception to 25th March 2024 as follows: (“pharmacog* W/4 test*“) AND (hospital* OR community OR “primary care*“) AND (implement* OR model OR care OR service OR guid*) AND (CYP2C19 OR clopidogrel). This search was then adapted and carried out through MEDLINE via OVID, EMBASE, CINHAL and PubMed from database inception to 25th March 2024 *(see* Tables S1–S4*for full search strategies*). The search on these databases included additional keywords such as: “care service”, “practice*”, “cardio*” and “program implementation”. No language or time limitations were applied to the search.

Relevant subject headings were coupled to the matching search concepts with “OR”. The key subject headings (MeSH) included: “pharmacogenetic testing”, “pharmacogenetics”, “genetic testing”, “clopidogrel”, “cytochrome P450 2C19”, “hospital service”, “health service”, “community”, “community hospital”, “community service”, “community outpatient service” and “hospital programs”.

### Source selection

Search results were imported into Covidence where duplicates were removed. Two independent reviewers (TJP, EW) then performed title and abstract screening, followed by full text screening for articles meeting the eligibility criteria. Eligible studies were then included for data extraction. A third reviewer (SLS) helped reach a final consensus where conflicts were unable to be resolved.

### Data items

An excel spreadsheet was developed to capture relevant data. The extracted data included: study details (e.g., year, country), clinical setting (e.g., hospital), sample size, methods of patient recruitment and identification of testing eligibility, staff or healthcare professionals involved in service delivery and their roles, training and/or education provided, sample type (e.g., buccal swab), laboratory location, result delivery framework, average turnaround time, process outcomes, barriers related to service implementation, and the pharmacogenomic genes tested (*see* Table S5).

### Data synthesis

Results were separated into two distinct tables and were grouped according to the healthcare setting in which the service was implemented. This was classified as either “hospital” or “community”. Testing that occurred in a medical centre (defined as an institution providing primary healthcare services with no distinct description of hospital features) or specialist clinic was classified as occurring in the “community”. Involvement of key healthcare professionals (HCPs) was recorded, and their roles were standardised to predefined terms and definitions (see Table 1) to enable comparison across the various services. The sample type was recorded as a “buccal swab”, “blood sample” or “saliva sample”. Sample analysis was recorded to occur either “internally” for laboratories reported to be on-site or where a point-of-care device was used, and “externally” for services requiring transport of the sample to an off-site laboratory. Where reported, the average turnaround time of the CYP2C19 genotype result was extracted, allowing comparison between time and location of sample analysis. Data on training and education was recorded as “yes” or “no” depending on whether any training/education programs were described, and whether they were mandatory or optional. The mode of training (e.g., online webinar) was also collected. Methods of result notification (e.g., physician notified via e-mail), and documentation (e.g., results integrated into the electronic health record (EHR)) were also summarised. Additionally, key process outcomes (e.g., physician acceptance rates) (see Table S6), and barriers relating to service implementation were also recorded.

**Table 1:** Definitions of key terms used to categorise the roles of staff and healthcare professionals within described services.

Term	Definition
Analysing	Examining lab results and their implications for the patient.
Approving	Approving and validating results prior to availability. Approving request for pharmacogenomic testing.
Consulting	Liaising with other HCP’s regarding treatment or results. Counselling patients on implication of results.
Documenting	Entering results into the database at the site of implementation/practice.
Ordering	Requesting for a pharmacogenomic test to be completed for a patient.
Recruiting	Recruiting and obtaining participant consent for a research study.
Referring	Directing a patient to a pharmacogenomic testing service to obtain a pharmacogenomic test.
Reporting	Generating reports/notes outlining clinical recommendations based on the pharmacogenomic lab results.
Reviewing	Examining recommendations and their clinical implications for the patient and their treatment.
Sampling	Collecting a blood and/or saliva sample, or a buccal swab.
Training	Educating involved personnel prior to involvement in the study.

## Results

### Source selection

**Fig. 1:**
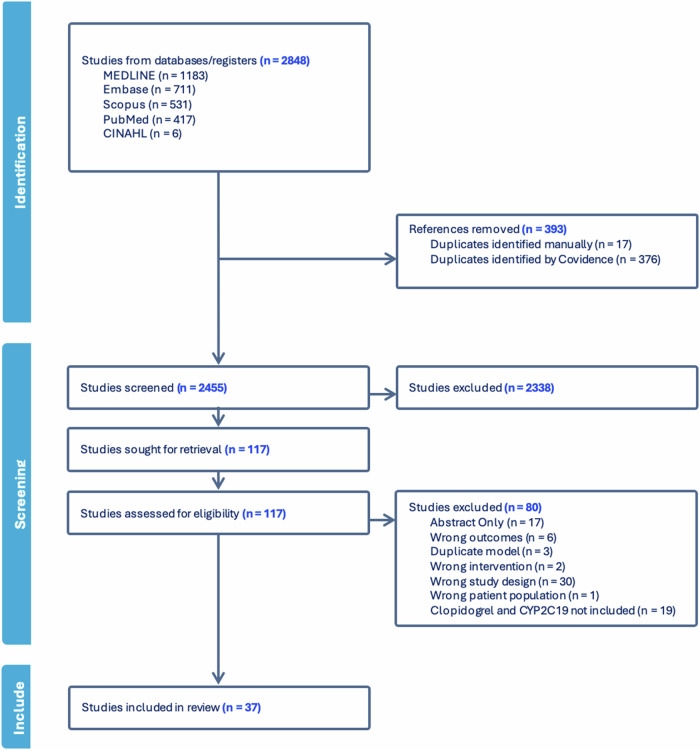
PRSIMA flow diagram of study selection.

The search retrieved a total of 2 848 studies, of which 2 455 were screened for their title and abstract following duplicate removal. Of these, 117 studies qualified for full text screening. Citation searching of three reviews was conducted to obtain original studies for full text screening [17–19], after which 37 were eligible for study inclusion and data extraction (see Fig. 1).

### Study characteristics

The characteristics of the included studies and key service components are summarised in Tables 2, 3. Most studies (22/37, 59%) described a service implemented in a hospital setting whilst 14 studies (38%) described one occurring in the community. One study described a service implemented across both settings [20]. Most services reported were from the United States (28/37, 76%). The utilisation of a panel testing approach (23/37, 62%) was more common than a single-gene test (14/37, 38%). One paper described two different testing services occurring in the same setting [21]. Methods of patient recruitment varied based on clinical setting and whether a panel test or single-gene test was utilised. Services in the community identified eligible patients primarily through physician referral or at the point-of-dispensing. Hospital services utilising a single-gene testing approach recruited patients undergoing PCI or identified those prescribed clopidogrel through the EHR. Hospitals utilising a panel testing approach recruited patients based on whether a drug with an actionable pharmacogenomic marker was prescribed on the EHR. Training and education was a core component in most services (22/37, 59%), with 17 studies describing mandatory training for HCPs involved in the delivery of the service (see Table 3). Training was commonly delivered prior to service implementation and through a variety of modes including in-person training, seminars and online webinars. Additionally, sample sizes were reported by all but 11 studies, of which 2 were planning papers and 9 provided only descriptions of services already implemented without reporting on patient outcomes.

**Table 2:** Summary of characteristics of included studies and components of their service model.

Study, Year	Country	Sample Size	Patient Eligibility/Recruitment	Sample Type	Laboratory Location	Average Result Turnaround Time	PGx Testing Approach
Community Based Pharmacogenomic Services							
O’Connor et al. [32]	USA	NR	Patients presenting with clopidogrel script for post PCI, ACS, TIA or stroke treatment	Buccal Swab	External	7 days	Single-gene
Ferreri et al. [54]	USA	18	Patients presenting with clopidogrel script	Buccal Swab	External	5.6 days	Single-gene
Haga et al. [24]	USA	NR	Patients with script/prescribed medication affected by included PGx markers	Saliva Sample	External	3–7 days	Panel
Bright et al. [37]	USA	30	Adult patients who underwent PCI in the last 12 months prescribed an antiplatelet and had no previous PGx testing completed	Buccal Swab	External	**Lab 1:** 1 day **Lab 2:** 3–5 days	Single-gene
Moaddeb et al. [46]	USA	56	Patients presenting with a prescription for clopidogrel or simvastatin	Buccal Swab	External	7 days	Panel
Dunnenberger et al. [33]	USA	76	Physician referral to the program	Buccal Swab	External	7 days	Panel
Papastergiou et al. [50]	Canada	100	Patients >18 yrs reporting adverse events, ineffective therapy or guiding initiation of therapy	Buccal Swab	External	14 days	Panel
Bain et al. [53]	USA	296	Patients >55 yrs enrolled in the PACE program that were not responding to drugs, prescribed a new drug or required reassessment	Buccal Swab	External	NR	Panel
Bright et al. [41]	USA	54	Patients prescribed a P2Y12 inhibitor	Buccal Swab	Internal (P.O.C.)	70 min	Single-gene
Dressler et al. [58]	USA	51	Patients taking a medication with actionable PGx guidelines invited to participate	Buccal Swab	External	NR	Panel
Van Der Wouden et al. [25]	Netherlands	NR	Patients presenting with script for one of the 39 DPWG PGx drugs	Saliva Sample	External	NR	Panel
Haga et al. [31]	USA	150	Patients presenting with prescription for medications affected by 1 of 5 included genes	Buccal Swab	External	NR	Panel
Levens et al. [47]	Netherlands	144	Patients taking either prasugrel or ticagrelor and have not undergone previous PGx testing	Buccal Swab	Internal (P.O.C)	75 min	Single-gene
Mir et al. [26]	Spain	16	Patients >18 yrs prescribed clopidogrel	Saliva Sample	External	13.8 days	Single-gene
Hospital Based Pharmacogenomic Services							
Johnson et al. [28]	USA	NR	Patients undergoing left-heart catheterisation	NR	External	NR	Single-gene
Hoffman et al. [34]	USA	1 559	Patients taking medications affected by included PGx markers	Blood Sample	External	20–137 days	Panel
Shuldiner et al. [38]	USA	NR	Patients undergoing cardiac catheterisation	Blood Sample	Internal	5 h	Single-gene
Cutting et al. [39]	USA	NR	Patients prescribed a medication leading to an EHR alert informing prescriber of the need for testing	Blood Sample	External	NR	Single-gene
Brixner et al. [59]	USA	205	Patient ≥65 yrs that had a dose change or initiated a medication from included medications in the last 120 days, and were on 3 or more medications	Buccal Swab	External	NR	Panel
Caraballo et al. [29]	USA	3 788	Patients who are prescribed a medication leading to an EHR alert	NR	Internal	NR	Panel
^a^Bergmeijer et al. [21] *Popular Risk Score Project*	USA	2 556	Patients undergoing PCI	Blood Sample	Internal + External	90% available < 24 h (median time = 4:04 h)	Single-gene
*Popular Genetics Study*	USA	1 038	Patients admitted with a STEMI or undergoing PCI		Internal	2:24 h (blood sample) 2:16 h (POC) 52 h (external lab)	Single-gene
Borobia et al. [30]	Spain	2 539	Patients taking medication affected by included PGx markers	Blood Sample	Internal	NR	Panel
Cavallari et al. [45]	USA	931	Patients with planned heart catheterisation with intent to undergo PCI	Buccal Swab	Internal	90 min	Single-gene
Di Francia et al. [35]	Italy	150	Patient with active cancer and/or frail cardiac admissions	Buccal Swab	External	7 days	Panel
Petry et al. [44]	USA	NR	Patients taking medications affected by included PGx markers	Blood Sample	Internal	NR	Panel
Christensen et al. [42]	USA	11 000	Patients within the health network invited to participate in the program	Blood Sample	Internal	14 days	Panel
Cohn et al. [22]	Canada	172	**P.O.C Cohort:** commenced a drug and/or had ADR/inadequate response to a drug **Pre-emptive:** children with cardiac disease	Buccal Swab Blood Sample	Internal (P.O.C)	NR	Panel
Gill et al. [43]	USA	NR	Prescriber orders directly through EHR for included drugs/markers	Blood Sample	Internal	NR	Panel
Liko et al. [52]	USA	NR	Referral from the physician to the program	Buccal Swab	External	10 days	Panel
Liu et al. [36]	USA	NR	Patients taking medication affected by included PGx markers	Blood Sample	Internal	NR	Panel
Russmann et al. [51]	Switzerland	56	Patients referred by physician if commencing or continuing clopidogrel therapy	Blood Sample	External	NR	Single-gene
Wang et al. [48]	USA	10 077	Volunteers who donated biospecimens to Mayo Clinic Biobank	Blood Sample	Internal	NR	Panel
Aquilante et al. [27]	USA	NR	Self-enrolling patients through an online portal	Blood Sample Saliva Sample	External	NR	Panel
Gurbel et al. [49]	USA	1 052	Patients undergoing PCI	Buccal Swab	Internal (P.O.C.)	1 h	Single-gene
Lteif et al. [40]	USA	99	Self-identifying Black and Latino patients	Buccal Swab	External	7 days	Panel
Voicu et al. [23]	Switzerland	167	Patients with indication for clopidogrel therapy	Buccal Swab Blood Sample	Internal (P.O.C)	<24 h (buccal swab) 1–2 weeks (blood sample)	Single-gene
Both Hospital and Community Based Service							
Al-Mahayri et al. [20]	United Arab Emirates	160	Adult patients prescribed clopidogrel, atorvastatin, rosuvastatin or warfarin	Blood Sample	External	24–48 h	Panel

Studies ordered chronologically according to year of publication.

*NR* not reported, *P.O.C*. point of care, *PCI* percutaneous coronary intervention, *EHR* electronic health record, *ADR* adverse drug reaction, *TIA* transient ischaemic attack, *DPWG* Dutch Pharmacogenetics Working Group, *PGx* pharmacogenomic, *STEMI* ST-elevation myocardial infarction.

^a^Study described two different testing services occurring in the same setting in the same paper.

**Table 3:** Summary of staff and healthcare professional involvement, pre-implementation training, result communication methods and barriers to implementation reported by included studies.

Study, Year	Key Staff and Practitioners Involved and Their Roles	Education/Training Provided	Result Notification & Documentation Framework	Reported Barriers to Service Implementation
Community Based Pharmacogenomic Services				
O’Connor et al. [32]	Pharmacist: Ordering, Analysing, Reporting, Consulting Physician: Reviewing, Documenting, Reporting, Consulting	Yes – Mandatory	• Pharmacist notified physician via fax. • Results incorporated into pharmacy record + available from online lab portal.	• Documenting results within the pharmacy EHR
Ferreri et al. [54]	Pharmacist: Sampling, Analysing, Reporting, Consulting Physician: Reviewing Laboratory: Sampling, Reporting	NR	• Pharmacist notified of results via e-mail. • Report with personalised recommendations faxed to physician.	• Inability to access full patient history
Haga et al. [24]	Pharmacist: Analysing, Consulting Physician: Ordering, Consulting	Yes – Mandatory	• Pharmacist and physician notified of results electronically from laboratory.	• NR
Bright et al. [37]	Study Investigators: Recruiting, Training. Pharmacist: Analysing, Recruiting, Sampling, Reporting, Consulting Physician: Referring, Reviewing	Yes – Mandatory	• Study investigators notified via e-mail. • Generated report with personalised recommendations.	• Inability to access full patient history • Limited testing scope of laboratory causing higher turnaround times
Moaddeb et al. [46]	Pharmacist: Recruiting, Sampling, Analysing, Reporting, Consulting Physician: Reviewing, Consulting	NR	• Pharmacist + physician faxed results from lab. • Results available from online lab portal.	• Lack of linked on-demand resources for clinicians on EHR
Dunnenberger et al. [33]	Geneticist: Consulting, Sampling Genetic Counsellor: Consulting Nurse Practitioner: Consulting Pharmacist: Consulting, Sampling, Analysing Physician: Ordering, Reviewing Laboratory: Reporting	Yes – Optional	• Physician notified via e-mail. • Patient handed results during consultation. • Results scanned into EHR.	• Physician unable to order PGx test through EHR • Lack of linked on-demand resources for clinicians on EHR
Papastergiou et al. [50]	Pharmacist: Sampling, Analysing, Consulting Laboratory: Sampling, Analysing, Reporting Physician: Consulting	Yes – Mandatory	• Pharmacist notified of results via online lab portal. • Patient handed results during pharmacist consultation.	• Incorporation into pre-existing workload • Receiving timely communication from prescriber
Bain et al. [53]	Pharmacist: Analysing, Consulting, Reporting Physician: Referring, Consulting, Reviewing Program Staff: Sampling	Yes – Mandatory	• Pharmacy e-mailed physician report with personalised recommendations. • Results documented in pharmacy records.	• Incorporation into pre-existing workflow • Documenting results within pharmacy EHR • Limited access to patient history
Bright et al. [41]	Technician: Recruiting, Sampling, Consulting Pharmacist: Analysing, Sampling, Reporting, Consulting Physician: Reviewing	Yes – Mandatory	• Physician sent report with personalised recommendations via e-mail.	• Incorporation into pre-existing workflow • P.O.C. device required run time increased patient wait time
Dressler et al. [58]	Pharmacist: Analysing, Reporting, Consulting Physicians: Reviewing, Consulting	Yes – Mandatory	• Physician + pharmacist notified of results via e-mail from lab. • Generated report with personalised recommendations.	• Lack of documentation into the EHR reducing physician accessibility
Van Der Wouden et al. [25]	Pharmacist: Recruiting, Sampling, Analysing, Reporting, Consulting, Documenting Physician: Consulting Laboratory: Reporting, Consulting	Yes – Mandatory	• Pharmacist notified of results via email/fax from lab. • Results documented into the EHR.	• Documenting within EHR time-consuming and error prone
Haga et al. [31]	Pharmacist: Recruiting, Sampling, Analysing, Consulting Physician: Approving	Yes – Mandatory	• Results returned to patient.	• Incorporation into pre-existing workflow
Levens et al. [47]	Pharmacist: Recruiting, Sampling, Analysing, Reporting, Consulting, Documenting Physician: Reviewing, Consulting	Yes – Mandatory	• GP/physician emailed or phoned results + sent report with recommendations. • Documented within pharmacy records.	• Increased administrative burden when results are not on EHR • Incorporating into existing workload
Mir et al. [26]	Pharmacist: Analysing, Recruiting, Sampling, Consulting Geneticist: Analysing, Reporting Physician: Reviewing	Yes – Mandatory	• Patients notified of results via pharmacist consult. • Documented into the pharmacy records.	• NR
Hospital Based Pharmacogenomic Services				
Johnson et al. [28]	PGx Committee: Approving, Training Cardiologists: Ordering Pharmacist: Analysing, Consulting	NR	• Physician and pharmacist notified via CDS alert. • Documented into EHR.	• Incorporating test ordering into pre-existing workflow
Hoffman et al. [34]	Nurse: Consulting Laboratory: Reporting Informaticist: Approving Pharmacist: Analysing, Reporting, Consulting, Reviewing Pathologist/Physician: NR	Yes – Optional	• Hospital notified electronically of results. Results sent as letters to consenting patients. • Documented into the EHR + integrated within CDS system. • Generated report with personalised recommendations.	• Result turnaround time from laboratory to hospital
Shuldiner et al. [38]	Study Investigators: Sampling, Consulting Lab Director: Approving, Analysing, Reporting Lab Staff: Consulting Pharmacist: Ordering, Reviewing Nurses/Genetic Counsellors/Physician: NR	Yes – Optional	• Physician notified either verbally through phone consult or e-mail/fax. • Results documented within the EHR + integrated into CDS system.	• Documenting results within EHR • Improper training of included staff
Cutting et al. [39]	Physician: Ordering Lab Director: Approving Lab Staff: Analysing, Consulting, Reporting Study Investigators: Reviewing, Reporting, Consulting	NR	• Physician notified of actionable results via pager. • Results relayed verbally to physician on phone. • Documented into the EHR. • Documented on patient’s paper chart.	• Documenting results within EHR at several points • Page-and-call method was untimely • Full report unavailable to clinician
Brixner et al. [59]	Pharmacist: Analysing, Reporting, Consulting Physician: Reviewing, Consulting	NR	• Physician notified via e-mail. • Generated report with personalised recommendations.	• NR
Caraballo et al. [29]	Pharmacist: Consulting Physicians/Assistants/Nurse Practitioners: NR Study Investigators: Training	Yes – Optional	• Documented into the EHR + integrated within CDS system. • Generated report with personalised recommendation.	• Documenting results within EHR. • Interpreting clinical guidelines
^a^Bergmeijer et al. [21] *Popular Risk Score Project*	Physician: Analysing Lab Technicians: Sampling	Yes – Mandatory	• Documented into the EHR.	• P.O.C. device only allowed 1 sample to be analysed concurrently • Expensive
*Popular Genetics Study*	Nurse: Sampling Program Staff: Sampling Physician/Pharmacist: NR	Yes – Mandatory	• Cardiologist, GP and pharmacist notified via e-mail. • Results documented into the EHR.	• Shipment of sample to external lab delaying results
Borobia et al. [30]	Geneticist: Reporting Pharmacologist: Reporting Physician: Reviewing	Yes – Mandatory	• Physician notified via EHR. • Results documented into the EHR.	• Determining who receives and benefits from reports
Cavallari et al. [45]	Pharmacist: Analysing, Reporting. Physician: Reviewing.	NR	• Physician notified via e-mail. • Documented into the EHR. • Integrated within CDS system.	• In some cases, delayed result availability impacted workflow and lack of pharmacist involvement led to improper therapy
Di Francia et al. [35]	Physician: Ordering, Reviewing Pharmacist: Analysing, Reviewing, Reporting, Consulting Geneticist: Analysing, Reporting, Consulting Lab Director: NR	NR	• Physician notified via laboratory	• Low patient recruitment due to inclusion criteria specificity
Petry et al. [44]	Lab Technologist: Reporting, Documenting Pharmacist: Analysing, Reporting, Consulting, Documenting Physician: NR	Yes – Mandatory	• Results documented into the EHR + integrated within CDS system.	• Documenting results into EHR
Christensen et al. [42]	Lab Director: Approving Genetic Counsellor: Approving, Consulting Pharmacist: Analysing, Reporting, Documenting Physicians: Ordering, Reviewing	Yes – Mandatory	• Physician notified via EHR. • Patients access results through online portal. • Generated report with personalised recommendations. • Documented into the EHR + integrated within CDS system.	• NR
Cohn et al. [22]	Pharmacist: Analysing, Consulting Patient Team: Reviewing Physician: NR	NR	• Physician & team members notified through EHR. • Patients + physician sent report with personalised recommendations.	• NR
Gill et al. [43]	Physician: Ordering, Reviewing Pathologist: Analysing, Reporting, Consulting, Documenting Pharmacist: NR	NR	• Physician notified via EHR. • Documented into the EHR + integrated within CDS system.	• Turnaround time • Documenting results into EHR • Lack of linked resources on EHR
Liko et al. [52]	Physician: Referring, Reviewing, Consulting Pharmacist: Sampling, Analysing, Reporting, Consulting	NR	• Pharmacist, physician, and patients notified via e-mail from lab. • Generated report with personalised recommendations. • Documented into the EHR.	• Pharmacist “on-demand” structure for PGx testing service not feasible
Liu et al. [36]	Laboratory Staff: Reporting, Documenting Pharmacist: Analysing, Reporting Geneticists/Physicians: NR	NR	• Patients notified + access results via online portal. • Documented into the EHR + integrated within CDS system.	• NR
Russmann et al. [51]	Physician: Referring Pharmacologist: Analysing, Consulting, Reporting Pharmacist: Reviewing	NR	• Physician and patient sent report with personalised recommendations electronically.	• NR
Wang et al. [48]	Pharmacist: Analysing, Reporting, Consulting Physicians: Reviewing	Yes – Mandatory	• Physician notified verbally through phone call. • Documented into the EHR+ integrated within CDS system.	• Translating patient PGx results into dosing recommendations
Aquilante et al. [27]	Geneticist: Approving Physician: Reviewing Pharmacist: Analysing, Reporting, Consulting	Yes – Optional	• Primary-care provider notified. • Documented into the EHR + integrated within CDS system.	• NR
Gurbel et al. [49]	Nurse: NR Physician: Ordering, Reviewing Pharmacist: Analysing	Yes – Mandatory	• Physician notified verbally of results. • Documented into the EHR.	• NR
Lteif et al. [40]	Study Investigators: Recruiting Pharmacist: Analysing, Documenting, Reporting Physician: NR	NR	• Physician notified via CDS alert. • Patient’s PCP emailed consult note by pharmacist. • Documented into the EHR + integrated within CDS system	• Turnaround time
Voicu et al. [23]	Senior Pharmacologist: Analysing, Reporting Research Team: Reporting, Documenting Physician: Sampling Nurse: Sampling	NR	• Physician sent report with personalised recommendations electronically.	• Lack of accessible guidelines to follow for result analysis
Both Hospital and Community Based Service				
Al-Mahayri et al. [20]	Physician: Analysing Pharmacist: NR	NR	• Physician notified via e-mail • Generated report with personalised recommendations.	• Incorporation into pre-existing workflow

*EHR* electronic health record, *L.o.F* loss of function, *NR* not reported.

^a^Study described two different testing services occurring in the same setting in the same paper.

### Sample type and site of laboratory

Buccal swabs (19/37, 51%) and blood collections (15/37, 41%) were the most common methods of patient sampling utilised. Of these, two studies reported using both a blood sample and buccal swab [22, 23]. Services within the community primarily utilised buccal swabs whilst blood samples were more common in the hospital. A saliva sample was an uncommon approach, reported by only four studies [24–27], of which one utilised both a blood and saliva sample [27]. Two studies did not report their sampling method [28, 29].

Sample analysis occurred externally for 23 studies and internally for 15 studies. One study used both laboratory locations [21]. Community based services generally sent samples to external laboratories (10/14, 71%) for analysis whilst hospital based services utilised an internal laboratory more frequently (14/22, 64%). A point-of-care approach was described by five studies (three community, two hospital), which all reported using a buccal swab for patient sampling.

### Average test result turnaround time

Twenty-one studies (21/37, 57%) reported a turnaround time for receiving the pharmacogenomic test result. The turnaround time of an external laboratory ranged from 1–14 days with results commonly becoming available within seven days, whilst an internal laboratory ranged from approximately two to five hours. The five studies utilising a point-of-care approach reported the quickest turnaround times, ranging from 70 min to 24 h, with results commonly being delivered within 90 min in most cases.

### Staff and healthcare professional involvement and their roles

Involvement of HCPs from a range of disciplines was reported. Most services involved a pharmacist (34/37, 92%), with one study utilising both a community and hospital pharmacist [26]. In two studies where pharmacist involvement was absent, a pharmacologist was involved in analysing, reporting, consulting, and reviewing results [23, 30]. For the services utilising a pharmacist, their main roles were to analyse, consult and report (see Table 4). They were involved in reviewing and ordering to a lesser extent and played no role in approving and referring. A small proportion of pharmacists were also involved in patient sampling across hospital and community services.

**Table 4:** Distribution of roles performed by physicians, pharmacists and laboratory staff as described within the pharmacogenomic testing services reviewed.

Roles Performed	Physicians (*n* = 37)	Pharmacists (*n* = 34)	Laboratory Staff (*n* = 13)
Analysing	2 (5%)	28 (82%)	3 (23%)
Approving	1 (3%)	0	3 (23%)
Consulting	10 (27%)	24 (71%)	3 (23%)
Documenting	1 (3%)	5 (15%)	2 (15%)
Ordering	8 (22%)	2 (6%)	0
Recruiting	0	6 (18%)	0
Referring	4 (11%)	0	0
Reporting	1 (3%)	20 (59%)	9 (69%)
Reviewing	20 (54%)	4 (12%)	0
Sampling	1 (3%)	11 (32%)	3 (23%)

Data reported as n (%). All pharmacogenomic services included physicians, 34 included pharmacists and 13 included laboratory staff.

Physicians played a key role in reviewing recommendations to guide treatment, and consulting with patients and other HCPs (most commonly the pharmacist). Since clopidogrel had already been initiated in most studies, physicians were primarily involved in altering treatment for individuals who were poor metabolisers. Physician approval for testing was required in one study [31] and documenting results into patient records within another [32]. They played no role in patient recruitment.

Nurse and/or nurse practitioner involvement was reported by seven studies, of which only four reported on their role. In these studies, they were involved in sampling [21, 23] and consulting [33, 34]. A geneticist was involved in six studies [26, 27, 30, 33, 35, 36] of which all but one [36] reported their role. They were involved in approving, sampling, analysing, reporting, and consulting. Additionally, direct involvement of study investigators in the service was reported by five studies [29, 37–40], with major roles in recruiting and training.

Other notable staff involvement included technicians [21, 41], physician assistants [29], laboratory directors [35, 38, 39, 42], pathologists [34, 43], genetic counsellors [33, 38] and a pharmacogenomics committee involved in training and overlooking program operations [28, 44].

### Result delivery and documentation framework

Electronic delivery and documentation of results was utilised amongst all but one service, which reported documentation on a paper chart [39]. Most services reported documenting results into the local EHR (25/37, 68%) through methods such as scanning [33] or manual data entry into pre-defined sections such as “contraindications” [25] or “laboratory results” [45]. Community based services primarily integrated pharmacogenomic results into pharmacy dispensing systems, whilst hospital based services integrated results into their local EHR. Of the hospital services utilising the EHR, 12 services (32%) reported integration into a clinical decision support (CDS) system which allowed for automatic detection of actionable CYP2C19 genotypes to help guide clinician decision making at the point-of-prescribing. Of these 12 studies, the majority (8/12, 67%) received results from an internal laboratory. To a lesser extent, CYP2C19 results were available through a laboratory-developed portal [32, 46]. A personalised report outlining treatment recommendations based on pharmacogenomic results was utilised by several services (14/37, 38%). One study attributed this as the reason behind observing a 57.5% prescriber acceptance rate in their service [30].

Notifying the corresponding HCP when results were available occurred primarily through email or fax (20/37, 54%). In some cases, physicians were notified verbally either in-person, or via phone call [38, 39, 47–49]. Patient notification was also reported through pharmacist or pharmacologist consultation [26, 33, 50, 51], online portal access [36, 42], e-mail from the laboratory [52] or a letter from study investigators [34]. Two studies did not report on the method of result notification [22, 31].

### Reported barriers of pharmacogenomic testing service

The incorporation of testing processes within pre-existing workflows was the most common barrier reported by clinicians involved within multiple services. This was commonly due to HCPs lacking time to perform the required tasks alongside their pre-existing responsibilities, and long physician response times to treatment recommendations, delaying treatment changes and patient consultations. Additionally, whilst the integration of results into the EHR was the most common method of documentation, it was not easily achieved as hospitals and community pharmacies reported lacking proper IT infrastructure [29, 38, 53], in addition to the processes being time-consuming and error-prone [25]. The issue of result portability and utility by other HCPs was also a reported barrier as once results were integrated into local EHR systems, a HCP involved in the care of patients external to the testing service could not access these results [39, 53]. Pharmacists who were particularly involved in analysing and reporting results indicated this lack of accessibility to be a major barrier in confidently outlining treatment recommendations [37, 53, 54]. Additionally, reports of physician inexperience in this field of practice led to result interpretation and patient consultation often being an arduous task when educational resources were not linked within the EHR [33, 43, 46].

By contrast, studies utilising a point-of-care approach reported barriers related to workflow incorporation [21, 41], sample accuracy [21] and testing costs [21, 47] rather than turnaround time.

## Discussion

This scoping review aimed to describe the range of pharmacogenomic testing services used to deliver CYP2C19 testing to guide clopidogrel therapy. Through this, several common elements were identified within the assessed studies. These included involvement of a multidisciplinary team of HCPs, electronic pathways for result communication and documentation, and buccal swabs or blood samples being the preferred sampling method. Additionally, whilst not reported by 15 studies, an education or training program appeared to be an important consideration, especially for community based services, of which all but 2 reported on.

A key focus of this review involved examining and describing the various roles of HCPs involved in the pharmacogenomic testing services reviewed. Through this, the need for a multidisciplinary team combining the activities of scientists, geneticists, pharmacists and physicians to interpret and apply pharmacogenomic information was identified. As expected, physician involvement was described within all services, and they played major roles in reviewing and consulting on recommendations. Additionally, within the multidisciplinary teams characterised, pharmacists were the second most commonly involved HCPs identified. They played a major role in analysing pharmacogenomic results and consulting with physicians to provide treatment recommendations. This is analogous to their role within other healthcare programs such as antimicrobial stewardship, indicating that there is potential to undertake a similar approach within the context of pharmacogenomics. Furthermore, pharmacist involvement was somewhat associated with higher prescriber acceptance rates, ranging from 63.1–100%, as compared to 57.5% reported by a study not involving a pharmacist [30]. However, data on this is limited and more robust research is required to confirm these preliminary findings. Nonetheless, based on the findings of this review, an imperative exists to involve pharmacists as part of a multidisciplinary team when designing a CYP2C19 service.

**Fig. 2:**
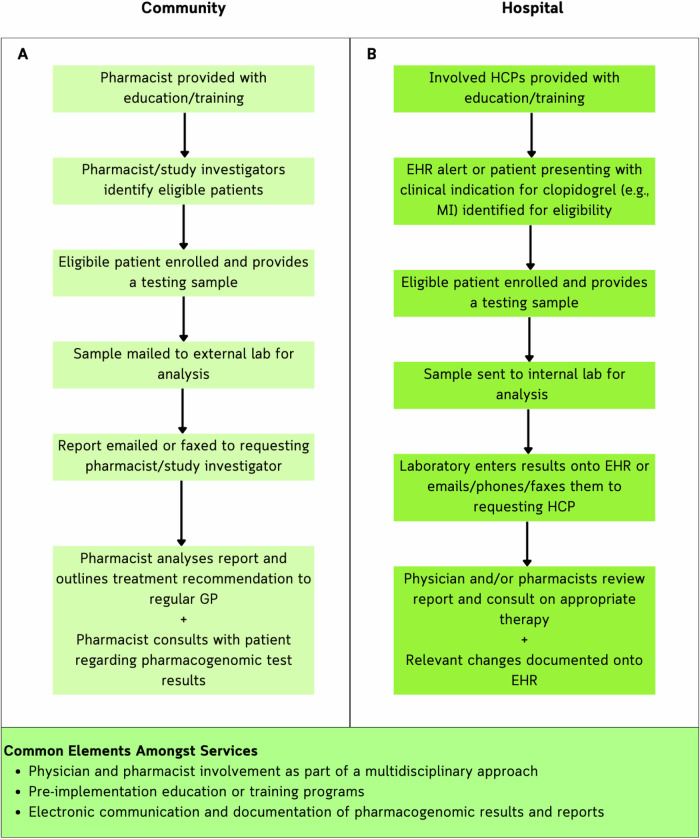
Comparison of the workflows described across hospital and community settings, with a summary of the common elements of a CYP2C19 testing service for clopidogrel relevant for both settings. In community-based services (**A**), pharmacists received pre-implementation training and education prior to study involvement. Once completed, they would recruit and obtain a sample from consenting patients to be mailed to an external laboratory for analysis. The laboratory would email or fax the results to the pharmacists, who would then analyse and generate a report outlining treatment recommendations for the GP and patients. They would then consult with the patient to explain their PGx results and their implications on treatment, and whether any treatment changes were to occur. In hospital-based services (**B**), numerous HCPs received training (e.g., clinicians, nurses, pharmacists) prior to study implementation. Patients were enrolled and tested if they had been, or were going to be, prescribed clopidogrel as indicated through an EHR alert. Internal labs analysed the sample and reported on the PGx results to the involved HCPs via email, fax or telephone. The PGx results would then be integrated into the patient's EHR. The clinician or pharmacist would then consult with the patient on their results and their implications on existing and future treatments.

Providing adequate training and education when implementing a new health service, such as a pharmacogenomic testing service, was reported by study authors as essential for improving clinician confidence, knowledge, and enhancing and maintaining the clinical uptake of pharmacogenomics [25, 37, 44]. Poor knowledge has been a commonly reported barrier preventing the routine implementation of pharmacogenomics within clinical practice [55], and one which needs to be addressed prior to service implementation. Training and education was delivered through a variety of modes including in-person lectures and seminars, online webinars and modules for involved HCPs to complete. No single educational approach was superior and so, training and education should ideally be designed to meet the needs of the healthcare institution and fill the knowledge gaps of the HCPs involved. Community based services primarily implemented mandatory training targeted towards pharmacists whilst in the hospital, training was more commonly optional and available to the wider multidisciplinary team. In both settings however, the training was provided prior to service implementation (see Fig. 2). This is especially important in guiding clopidogrel therapy as inappropriate treatment recommendations resulting from inadequate pharmacogenomic understanding could potentially lead to detrimental patient outcomes. More novel approaches for educating HCPs were also outlined, including imbedding external weblinks to educational resources within the EHR for HCPs to access when analysing and reviewing test results. This approach was deemed effective by one study [29], and preferred or requested by HCPs when it was not implemented by other studies [33, 43, 46], highlighting the potential role it could play in fostering positive outcomes.

Additionally, whilst not specific to guiding clopidogrel therapy alone, it is also important to consider how the CYP2C19 test results will be communicated and documented. In this review, results were communicated primarily via email or fax, providing the advantage of quick and easy communication amongst the multidisciplinary team. Services within hospitals primarily documented results into the EHR and CDS systems whilst those in the community reported documentation within the patient profile in the pharmacy dispensing system. However, as establishments often have local electronic/dispensing systems, there is an issue of result portability across transitions of care as test results may not be relayed to other HCPs. Additionally, the lack of standardised reporting of pharmacogenomic data further limits the widespread use of results across healthcare settings [56]. This is a major issue in many countries as the majority lack nationwide single patient health records and standardised data reporting, limiting the practical uptake of pharmacogenomics beyond the research environment. However, there have been approaches taken internationally to address this barrier. For example, the Netherlands utilises a nationwide CDS system where physicians and pharmacists are automatically notified of pharmacogenomic contraindications, if available, when prescribing or dispensing [25]. When utilised as part of the PREPARE trial, pharmacists and physicians reported benefits to having access to this system and the ability to access records in one place, alongside the DPWG guidelines imbedded within the program to help guide decision-making [25]. Whilst there is no data currently available on the clinical impact of this system, other similar strategies within US based services have reported promising results in fostering positive patient outcomes [57]. Additionally, the Pharmacogenomics Working Group at the Global Alliance for Genomics and Health is also aiming to develop a standardised approach for reporting pharmacogenomic data in order to improve the interoperability of pharmacogenomic results across health systems. Therefore, electronic result delivery and documentation, ideally within existing EHRs, with the potential to further integrate within CDS systems and single nationwide health records in the future, can help improve the uptake and applicability of pharmacogenomic data beyond the research landscape.

There were a few limitations of this review. Studies not explicitly indicating CYP2C19 and clopidogrel to be a gene-drug pair of focus were excluded, leading to descriptions of these services being missed. Majority of the papers reviewed described services within research-based settings rather than routine clinical practice. As a result, differences in service design and processes between these two settings could not be ascertained. Future research focussed on understanding the implementation of services part of routine clinical practice is required to gain real-world insights. Additionally, whilst a core focus of this review was detailing the HCPs and their roles, insight into how these roles were performed (e.g., how physicians ordered pharmacogenomic tests) was not possible as this level of detail was rarely provided in the studies reviewed. For the same reason, although majority of the studies integrated results with the EHR, the format in which they were integrated (e.g., PDF) could not be identified. Therefore, future research should aim to understand how to best perform the key processes required to facilitate an effective CYP2C19 service.

## Conclusion

In conclusion, this review identified that a multidisciplinary approach involving physicians and pharmacists, electronically conducted result delivery and documentation pathways and adequate training and education programs are common elements of CYP2C19 testing service for clopidogrel across both hospital and community settings. Integrating results within CDS systems and development of nationwide single patient health records has the potential to further the practical application of pharmacogenomics within practice. Thus, institutions looking to implement a CYP2C19 testing service to guide clopidogrel therapy may use the findings of this review as a basis for building and designing their service.

## Supplementary information

Supplementary Information

## Data Availability

The datasets generated during and/or analysed during the current study are available from the corresponding author on reasonable request.
