# Supplementary material for: Implementing CYP2C19-guided clopidogrel therapy: a scoping review of pharmacogenomic testing services
Source: Pharmacogenomics J. 2025 Apr 25;25(3):12. doi: 10.1038/s41397-025-00371-4 (PMC12031670; doi:10.1038/s41397-025-00371-4)
Supplement: Supplementary file 1 — Supplementary Information [file 41397_2025_371_MOESM1_ESM.docx]

**Supplementary Materials**

**Table S1 –** Search Strategy from Medline via OVID

| 1. | Pharmacogenetics/ or Pharmacogenomic Testing/ or Genetic Testing/ |
| --- | --- |
| 2. | pharmacogen*.mp. |
| 3. | test*.mp. |
| 4. | 2 and 3 |
| 5. | hospital*.mp. |
| 6. | community.mp. |
| 7. | primary care.mp. or Primary Health Care/ |
| 8. | 5 or 6 or 7 |
| 9. | implement*.mp. |
| 10. | strateg*.mp. |
| 11. | model*.mp. |
| 12. | 9 or 10 or 11 |
| 13. | 1 or 4 |
| 14. | 8 and 12 and 13 |

**Table S2 –** Search Strategy from EMBASE

| 1. | (pharmacogen* test* or pharmacogen* or test* or "pharmacogenetic testing" or "genetic testing").mp. |
| --- | --- |
| 2. | (implement* or strategy or program or "health service" or "testing service" or "health care service").mp. |
| 3. | (hospital or community or "primary care").mp. |
| 4. | pharmacogenetics/ or pharmacogenetic testing/ |
| 5. | clopidogrel/ |
| 6. | 1 or 4 |
| 7. | (clopidogrel or cyp2c19 or antiplatelet).mp. [mp=title, abstract, heading word, drug trade name, original title, device manufacturer, drug manufacturer, device trade name, keyword heading word, floating subheading word, candidate term word] |
| 8. | 5 or 7 |
| 9. | 1 and 2 and 3 and 8 |
| 10. | 2 and 3 and 6 and 8 |

**Table S3 –** Search Strategy from CINHAL

|  | (MH "Pharmacogenetics") |
| --- | --- |
|  | (MH "Program Implementation") OR (MH "Systems Implementation") |
|  | (MH "Hospitals, Community") OR (MH "Hospitals+") OR (MH "Communities+") OR (MH "Outpatient Service") OR (MH "Community Service") OR (MH "Community Programs") OR (MH "Hospital Programs") |
|  | (MH "Individualized Medicine") |
|  | S1 OR S4 |
|  | "pharmacogenomic testing" |
|  | S1 OR S6 |
|  | S2 AND S3 AND S7 |
|  | S3 AND S6 |

**Table S4 –** Search Strategy from PUBMED

|  | Pharmacogen* AND test* |
| --- | --- |
|  | Hospital OR Community OR “primary care” |
|  | Model OR implement* OR design OR “program implementation” OR strateg* OR service |
|  | Clopidogrel OR CYP2C19 OR cardio* |
|  | 1 AND 2 AND 3 AND 4 |

**Table S5 –** Complete list of genes tested for within the included studies.

| Study, Year | PGx Genes Tested |
| --- | --- |
| Al-Mahayri et al., 2022 | CYP2C19, VKORC1, CYP2C9, SLCO1B1, CYP4F2 |
| Aquilante et al., 2024 | CYP2C19, CYP2C9, SLC01B1, DPYD, TPMT, ABCG2, NUDT15 |
| Bain et al., 2018 | CYP2C19, CYP2C9, CYP2D6, CYP3A4, CYP3A5, CYP4F2, VKORC1, SLCO1B1, TPMT, ATM, F5 |
| Bergmeijer et al., 2018 | CYP2C19 |
| Borobia et al., 2018 | CYP2C19, CYP2D6, CYP3A4, CYP3A5, CYP4F5, TPMT, ABCB1, POR, VKORC1, SLCO1B1, UGT1A1, DPYD, TP53, APOE |
| Bright et al., 2015 | CYP2C19 |
| Bright et al., 2018 | CYP2C19 |
| Brixner et al., 2016 | CYP2D6, CYP2C9, CYP2C19, CYP3A4, CYP3A5, VKORC1 |
| Caraballo et al., 2017 | CYP2D6, CYP2C19, TPMT, HLA-B*5701, IL28B, HLA-B*1502, SLCO1B1, HLA-B*5801, VKORC1, CYP3A4, DYPD |
| Cavallari et al., 2018 | CYP2C19 |
| Christensen et al., 2021 | CYP2C9, CYP2C19, CYP2D6, CYP3A5, VKORC1, CYP4F2, DPYD, TPMT, SLCO1B1, IFNL3 |
| Cohn et al., 2021 | CYP2C19, CYP2C9, CYP2D6, CYP3A5, TPMT, VKORC1 |
| Cutting et al., 2015 | CYP2C19 |
| Di Francia et al., 2019 | CYP1A2, CYP2B6, CYP2C8, CYP2C9, CYP2C19, CYP2D6, CYP3A4, CYP3A5, DYPD, TYMS, MTHFR, GSTP1 |
| Dressler et al., 2019 | CYP2D6, CYP2C19, HLA-B*5801, CYP2C9, VKORC1, HLA*B-1502, HLA-B*5701, TPMT, IFNL3, CYP3A5, NUDT15, UGT1A1, CYP2B6, HLA-A*3101, DPYD, SLCO1B1 |
| Dunnenberger et al., 2016 | Drugs with CPIC guidelines at time of conducted research. |
| Ferreri et al., 2013 | CYP2C19 |
| Gill et al., 2021 | MT-RNR1, CYP2C19, CYP2D6, UGT1A1, TPMT, NUDT15, CYP2C9, ACYP2, F5, DDYD, ADRB2, NA, CYP3A4, SLCO1B1, CYP3A5, CEP72, VKORC1 CYP2C, DYP4F2 |
| Gurbel et al., 2021 | CYP2C19 |
| Haga et al., 2014 | CYP2D6, CYP2C19, HLA-B*1502, CYP2C9, SLCO1B1, VKORC1 |
| Haga et al., 2021 | CYP2C19, CYP2C9, CYP2D6, VKORC1, SLCO1B1 |
| Hoffman et al., 2014 | CYP2D6, CYP2C19, TPMT, SLCO1B1 |
| Johnson et al., 2013 | CYP2C19 |
| Levens et al., 2023 | CYP2C19 |
| Liko et al., 2021 | CYP1A2, CYP2B6, CYP2C9, CYP2C19, CYP2D6, CYP3A4/5, CYP4F2, COMT, DPYD, F2, F5, G6PD, GRIK4, HLA-A*3101, HLA-B*1502, HLA-B*5701, HLA-B*5801, HTR2A, HTR2C, IFNL4, NUDT15, OPRM1, SLC6A4, SLCO1B1, TPMT, UGT1A1, VKORC1 |
| Liu et al., 2021 | CYP2C19, SLCO1B1, VKORC1, TPMT, CYP2D6, CYP3A5 |
| Lteif et al., 2024 | CYP2C19, CYP2C9, CYP2D6, CYP3A5, CYP4F2, SLCO1B2, VKROC1, CYP2C cluster |
| Mir et al., 2023 | CYP2C19 |
| Moaddeb et al., 2015 | CYP2C19, SLCO1B1 |
| O'Connor et al., 2012 | CYP2C19 |
| Papastergiou et al., 2017 | CYP1A2, CYP2C9, CYP2C19, CYP2D6, CYP3A4, CYP3A5, OPRM1, SLCO1B1, VKORC1 |
| Petry et al., 2019 | CYP2C19, CYP2C9, CYP2D6, CYP3A5, DPYD, SLCO1B1, TPMT, VKORC1 |
| Russmann et al., 2021 | CYP2C19 |
| Shuldiner et al., 2014 | CYP2C19 |
| Van Der Wouden et al., 2020 | CYP2B6, CYP2C9, CYP2C19, CYP2D6, CYP3A5, DPYD, F5, HLA-B*5701, SLCO1B1, TPMT, UGT1A1, VKORC1 |
| Voicu et al., 2024 | CYP2C19 |
| Wang et al., 2022 | CYP2C19, CYP3A5, CYP2D6, CYP2C9, TPMT, DPYD, VKORC1, NUDT15, HLA-A*3101, HLA-B*1502, HLA-B*5701, HLA-B*5801, SLCO1B1 |

**Table S6 –** Reported process outcomes of included studies

| Study, Year | Process Outcomes |
| --- | --- |
| O'Connor et al., 2012 [22] | NR |
| Ferreri et al., 2014 [23] | 100% prescriber acceptance rate |
| Haga et al., 2014 [24] | NR |
| Bright et al., 2015 [25] | 24.1% of IM and UR metabolisers reported |
| Moaddeb et al., 2015 [26] | 94% of CYP2C19 results and 88% of SLCO1B1 results correctly interpreted |
| Dunnenberger et al., 2016 [27] | NR |
| Papastergiou et al., 2017 [28] | 17.6% of clopidogrel Rx required intervention; 63.2% physician acceptance rate |
| Bain et al., 2018 [29] | 89% acceptance rate on 436 pharmacist recommendations |
| Bright et al., 2018 [30] | Consultation + sampling required <10 min of pharmacist time |
| Dressler et al., 2019 [31] | 97% of participants exhibited at least 1 genetic variation |
| Van Der Wouden et al., 2020 [32] | Pharmacists reported satisfaction and confidence in service provision |
| Haga et al., 2021 [33] | NR |
| Levens et al., 2023 [34] | 100% of involved pharmacists response to post-study surveys.  96.5% of successful DNA swabs collected on first-try |
| Mir et al., 2023 [35] | No PGx-based treatment changed reported |
| Johnson et al., 2013 [36] | NR |
| Hoffman et al., 2014 [37] | NR |
| Shuldiner et al., 2014 [38] | NR |
| Cutting et al., 2015 [39] | NR |
| Brixner et al., 2016 [40] | Automated sample analysis reduced pharmacist burden of time |
| Caraballo et al., 2017 [41] | NR |
| *Bergmeijer et al., 2018 [21]  *Popular Risk Score Project* | NR |
| *Popular Genetics Study* | NR |
| Borobia et al., 2018 [42] | 57.5% physician acceptance rate.  Individualised reports improved physician acceptance |
| Cavallari et al., 2018 [43] | Clinical pharmacy support led to higher clopidogrel escalation rates |
| Di Francia et al., 2019 [44] | NR |
| Petry et al., 2019 [46] | NR |
| Christensen et al., 2021 [46] | NR |
| Cohn et al., 2021 [47] | 3 of 5 patients tested for CYP2C19 experienced cardiac event due to clopidogrel therapeutic failure |
| Gill et al., 2021 [48] | NR |
| Liko et al., 2021 [49] | NR |
| Liu et al., 2021 [50] | NR |
| Russmann et al., 2021 [51] | 71% physician acceptance rate |
| Wang et al., 2022 [52] | NR |
| Aquilante et al., 2024 [53] | NR |
| Gurbel et al., 2024 [54] | 93% physician acceptance rate within PCI recommendations |
| Lteif et al., 2024 [55] | NR |
| Voicu et al., 2024 [56] | 75.9% acceptance rate on recommendations for L.o.F allele carriers. 83% acceptance rate of clopidogrel therapy escalation in high-risk patients |
| Al-Mahayri et al., 2022 [20] | 38.1% intermediate metabolisers and 25% rapid/ultra-rapid metabolisers characterised |
